# Autophagy Mediates the Delivery of Thrombogenic Tissue Factor to Neutrophil Extracellular Traps in Human Sepsis

**DOI:** 10.1371/journal.pone.0045427

**Published:** 2012-09-19

**Authors:** Konstantinos Kambas, Ioannis Mitroulis, Eirini Apostolidou, Andreas Girod, Akrivi Chrysanthopoulou, Ioannis Pneumatikos, Panagiotis Skendros, Ioannis Kourtzelis, Maria Koffa, Ioannis Kotsianidis, Konstantinos Ritis

**Affiliations:** 1 First Department of Internal Medicine, Democritus University of Thrace, University General Hospital of Alexandroupolis, Alexandroupolis, Greece; 2 Life Sciences Research Unit-FSTC, University of Luxembourg, Walferdange, Luxembourg; 3 Intensive Care Unit, Democritus University of Thrace, University General Hospital of Alexandroupolis, Alexandroupolis, Greece; 4 School of Biology, Aristotle University of Thessaloniki, Thessaloniki, Greece; 5 Laboratory of Cellular and Molecular Biology, Department of Molecular Biology and Genetics, Democritus University of Thrace, Alexandroupolis, Greece; 6 Department of Hematology, Medical School, Democritus University of Thrace, Alexandroupolis, Greece; University of Bern, Switzerland

## Abstract

**Background:**

Sepsis is associated with systemic inflammatory responses and induction of coagulation system. Neutrophil extracellular traps (NETs) constitute an antimicrobial mechanism, recently implicated in thrombosis *via* platelet entrapment and aggregation.

**Methodology/Principal Findings:**

In this study, we demonstrate for the first time the localization of thrombogenic tissue factor (TF) in NETs released by neutrophils derived from patients with gram-negative sepsis and normal neutrophils treated with either serum from septic patients or inflammatory mediators involved in the pathogenesis of sepsis. Localization of TF in acidified autophagosomes was observed during this process, as indicated by positive LC3B and LysoTracker staining. Moreover, phosphatidylinositol 3-kinase inhibition with 3-MA or inhibition of endosomal acidification with bafilomycin A1 hindered the release of TF-bearing NETs. TF present in NETs induced thrombin generation in culture supernatants, which further resulted in protease activated receptor-1 signaling.

**Conclusions/Significance:**

This study demonstrates the involvement of autophagic machinery in the extracellular delivery of TF in NETs and the subsequent activation of coagulation cascade, providing evidence for the implication of this process in coagulopathy and inflammatory response in sepsis.

## Introduction

Systemic activation of coagulation cascade and formation of thrombi in the microvasculature contribute to organ dysfunction that characterizes sepsis [1], [2]. Several studies in experimental models indicate that septic inflammatory environment induces the expression of tissue factor (TF), a trans-membrane protein [2] which initiates coagulation cascade and results in thrombin generation. Circulating TF, in the form of TF-bearing microparticles or TF expressed in blood cells, is suggested to play a critical role in this process [1]–[5]. Additionally, thrombin and TF/factor VIIa complex signaling through protease activated receptor-1 (PAR-1) and PAR-2 respectively was implicated in the induction of inflammation in experimental models of sepsis, linking coagulation to inflammation [6], [7].

Neutrophils have a critical role in launching the first line of host defense against infection. They are recruited in vast numbers to the site of microbial invasion, where they engulf and kill pathogens into phagosomes [8], [9]. Moreover, neutrophils release lytic enzymes for the elimination of extracellular microbes [10]. Recently, another aspect of neutrophil microbicidal activity has been described; the release of neutrophil extracellular traps (NETs; [10], [11]). NETs are extracellular chromatin structures that entrap microbes and are composed of nuclear and granule constituents of neutrophils [10], [11]. They are formed after phagocytosis of pathogens or treatment with inflammatory stimuli [10], [11] and are implicated in the pathogenesis of sepsis [12]. Interestingly, it was shown that the entrapment of platelets in NETs is associated with platelet activation and aggregation [13]. Moreover, the presence of NETs has been recently indentified in thrombi in a murine model of deep vein thrombosis [14]. Experimental data further implicate neutrophil serine proteases in the inactivation of antithrombin and tissue factor pathway inhibitor which results in the activation of coagulation [15].

Several lines of evidence support a critical role for autophagy in the regulation of innate immune responses [16], [17]. This process has been implicated in pathogen elimination and recognition by intracellular receptors [16], [17]. The activation of autophagy in human neutrophils has been previously linked with phagocytosis and activation of Toll-like receptors [18]. Moreover, it is reported that neutrophils from patients with sepsis exhibit vacuolization and susceptibility toward a necrotic form of cell death, both associated with autophagy [19]. Additionally, recent data suggest that autophagy is required in NET release [20], [21].

Herein, we describe a novel role of neutrophils in the interface between inflammation and coagulation. We report for the first time that TF-bearing NETs are released from neutrophils derived from patients with gram-negative sepsis, trigger thrombin generation and subsequent PAR-1 signaling *in vitro*. Furthermore, inclusion of TF in autophagosomes was linked with its extracellular delivery in NETs, while inhibition of phosphatidylinositol 3-kinase (PI3K) signaling or endosomal acidification in neutrophils abolished NET release and TF trafficking.

## Materials and Methods

### Human Sepsis Neutrophil and Serum Samples

Peripheral blood neutrophils and serum were collected from six healthy donors and eight patients with gram-negative sepsis, hospitalized in First Department of Internal Medicine or Intensive Care Unit of University Hospital of Alexandroupolis. Informed written consent has been obtained from every volunteer or their relatives. The study protocol design was in accordance with the Declaration of Helsinki and the procedures have been approved by the local ethics committee (Scientific Committee of the University Hospital of Alexandroupolis, Greece). Patient characterization was in accordance to current clinical practice guidelines [22], [23]. The identification of gram-negative bacteria (*Escherichia coli* in six patients, *Pseudomonas aeruginosa* and *Klebsiella pneumonia* in the other two) from blood cultures obtained at the time of blood collection was required for the final inclusion of the experimental data in the study. Serum from all patients was obtained at the time of enrollment. Serum was immediately transferred to ice and isolated by centrifugation at 4°C at 1400×g for 15 min. Serum was stored at −80°C till used. Neutrophils were isolated from heparinized blood after double-gradient density centrifugation (Histopaque; Sigma-Aldrich Co., St Louis, MO, USA) as previously described [18]. Neutrophil purity (>98%) was assessed by Giemsa staining and viability (>95%) by Trypan blue staining. Contamination with platelets was under 1%. The absolute number of neutrophils was adjusted approximately to 4×10^6^ cells/ml in RPMI. After collection, all patient samples were numbered and de-identified for patient confidentiality.

### Stimulation and Inhibition Studies

Neutrophils were incubated in 5% CO_2_ at 37°C in a total volume of 500 µl of RPMI in the presence of 6% serum from healthy donor. Neutrophils were treated with either opsonized *Escherichia coli* (*E.* coli) at 1/30 neutrophil to bacteria ratio (Phagoburst, Phagotest; ORPEGEN Pharma, Heidelberg, Germany) or septic serum at a final concentration of 6% in neutrophil cultures for 3 h. This concentration was the optimal to stimulate neutrophils and avoid NET degradation. Moreover, 3 hours incubation with sepsis serum demonstrated the highest percentage of NET releasing neutrophils and thus this time point was chosen for further experiments. Cells were treated with the autophagy inhibitor 3-methyladenine (3-MA) (5 mM, Calbiochem, San Diego, CA, USA) or bafilomycin A1 (30 nM, Sigma-Aldrich Co.) 30 min after the addition of bacteria in order to allow phagocytosis to occur. Moreover, healthy neutrophils were incubated with a mixture of inflammatory mediators (TNF-α, 1 ng/ml, Sigma-Aldrich; G-CSF, 1 ng/ml, GRANOCYTE-Aventis, Antony, France; IL-1β, 10 ng/ml, Sigma-Aldrich). An IgG1 mouse anti-human TF mAb (10 µg/ml; American Diagnostica, Greenwich, CT, USA) was used for TF inhibition studies and an IgG1 anti-CD19 mAb (DAKO, Denmark) as control. PAR-1 signaling inhibition was performed using FLLRN peptide (500 µΜ, Anaspec, Fremont, CA, USA). TLR4 signaling inhibition was performed by pre-incubating sepsis serum with Polymyxin B (10 µg/ml, Santa Cruz, CA, USA) for 30 mins. All the substances used in this study were endotoxin free, as determined by a *Limulus* amebocyte assay (Sigma-Aldrich).

### Preparation of Apoptotic Neutrophils

Apoptotic neutrophils were prepared as previously described [24]. After incubation of neutrophils for 1 h with sepsis serum, apoptosis was induced by ultraviolet irradiation exposure (245 nm) for 10 min. Cells remained in RPMI with control serum in 5% CO_2_ at 37°C for 2 h. Apoptotic cells indicated more than 80% Annexin V positive and propidium iodide negative staining as assessed by flow cytometry (BD Biosciences, FACScan).

### Microparticle Depletion

Microparticles were depleted from sepsis serum as previously described [25]. The collected serum containing microparticles was centrifuged at 20,000×g for 15 minutes at 4°C to pellet them. Microparticle-depleted serum (supernatant) was centrifuged again at 20,000×g for 15 minutes at 4°C in order to eliminate remaining microparticles. Depletion of microparticles was verified by flow cytometry as previously described [26].

### Immunofluorescence

Sample preparation and visualization by immunofluorescence microscopy was performed as previously described [21]. Cells were stained using an anti-myeloperoxidase (MPO)-specific mouse monoclonal antibody (DAKO), an IgG1 mouse anti-TF mAb (American Diagnostica, Greenwich, CT, USA), a rabbit anti-LC3B polyclonal Ab (Sigma-Aldrich Co.) and a mouse anti-high mobility group box-1 (HMGB1) mAb (Abcam, UK). An IgG1 anti-CD19 mAb (DAKO) was utilized as control. A polyclonal rabbit anti-mouse Alexa fluor 488 antibody (Invitrogen, Carlsbad, CA, USA) was utilized as secondary antibody. DNA was counterstained using DAPI (Sigma-Aldrich). LC3B was stained with a goat anti-rabbit Alexa fluor 647 antibody (Invitrogen). LysoTracker (100 nM, Invitrogen) was used for the detection of acidified endosomes. Cell preparations were visualized in fluorescence microscope (Leica DM2000) or confocal microscope (Spinning Disk Andor Revolution Confocal System, Ireland) in a PLAPON 60×O/TIRFM-SP, NA 1.45 and UPLSAPO 100XO, NA 1.4 objectives (Olympus). The percentage of NET releasing cells was determined by the evaluation of 200 cells in a double-blind experimental procedure.

### NET Isolation

For protein detection/quantification on NETs, NET proteins were purified using DNase treatment and precipitated as previously described [21], [27]. NET structure isolation was performed by vigorous pippetting and sequential centrifugation, as previously described [28].

### Western Blot Analysis

For TF detection, western blotting was performed in precipitated samples as previously described [29]. A rabbit anti-human polyclonal ab (Santa Cruz) was used for TF detection and a rabbit anti-human polyclonal ab (Trevigen, MD, USA) for GAPDH. A goat anti-rabbit HRP conjugated polyclonal ab (R&D Systems, MN, USA) was used as secondary. To evaluate the expression of TF in NETs, equal amounts of total protein from each sample were loaded onto the gel to eliminate a potential discrepancy, due to the absence of a reference protein. For p62/SQSTM1 detection a mouse anti-human monoclonal ab (Santa Cruz) was used.

### TAT Complex ELISA Assay

Thrombin generation was studied in culture supernatants from healthy and neutrophils from patients with sepsis, treated for 3 h. Culture supernatants were collected and centrifuged for 10 min at 300×g. In the case of healthy neutrophils cultured *in vitro* in the presence of sepsis serum, culture medium was removed after 1 h of incubation, cells were washed twice with 1 ml pre-warmed RPMI and incubated for 2 h in fresh culture medium contained 6% serum from healthy donor. Considering that sepsis serum contains increased levels of thrombin, this step ensured the measurement of only NET-dependent thrombin generation. Afterwards, culture supernatants were collected and centrifuged at 300×g to remove any cells and at 16000×g to eliminate cellular debris. Isolated NETs structures were added in RPMI with 6% serum for 30 min. The concentration of thrombin-antithrombin (TAT) complex was measured according to manufacturer’s instructions (Assaypro, Winfield, MO, USA). The minimum detectable dose of the assay was 300 pg/ml.

### Platelet Activation Studies

Platelets were isolated from platelet rich plasma obtained from adult healthy donors as previously described [30], were suspended in HEPES buffer (200 µl) and incubated with the respective stimuli for 30 min at 37°C. Afterwards, 100 µl of these suspensions were stained with either anti-CD62P-PE antibody (Beckman Coulter, Switzerland) or annexin V-FITC antibody (BD Biosciences). Platelets were immediately diluted in 500 µl of HEPES buffer and analyzed by flow cytometry (BD Biosciences, FACScan). Analyses were performed on 10000 gated platelets in Cell Quest program (BD Biosciences).

### RNA Isolation, cDNA Synthesis and Quantitative Real-time PCR

RNA isolation, cDNA synthesis and TF qRT-PCR (primers and conditions) were performed as previously described [29]. Relative expression levels were determined using the 2^DDCT^ method.

### Statistical Analysis

Values are presented as mean ± SD. Mann-Whitney U-test or Wilcoxon test was used to assess the statistical significance of unpaired or paired samples (n≥6), respectively. For samples with n≥3 a Student’s t test was used. All statistical analyses were performed with GraphPad Prism (GraphPad Software, Inc., San Diego, CA). *P* values of ≤.05 were considered significant.

## Results

### Septic Inflammatory Environment Induces NET Release in an Autophagy-dependent Manner

**Figure 1:**
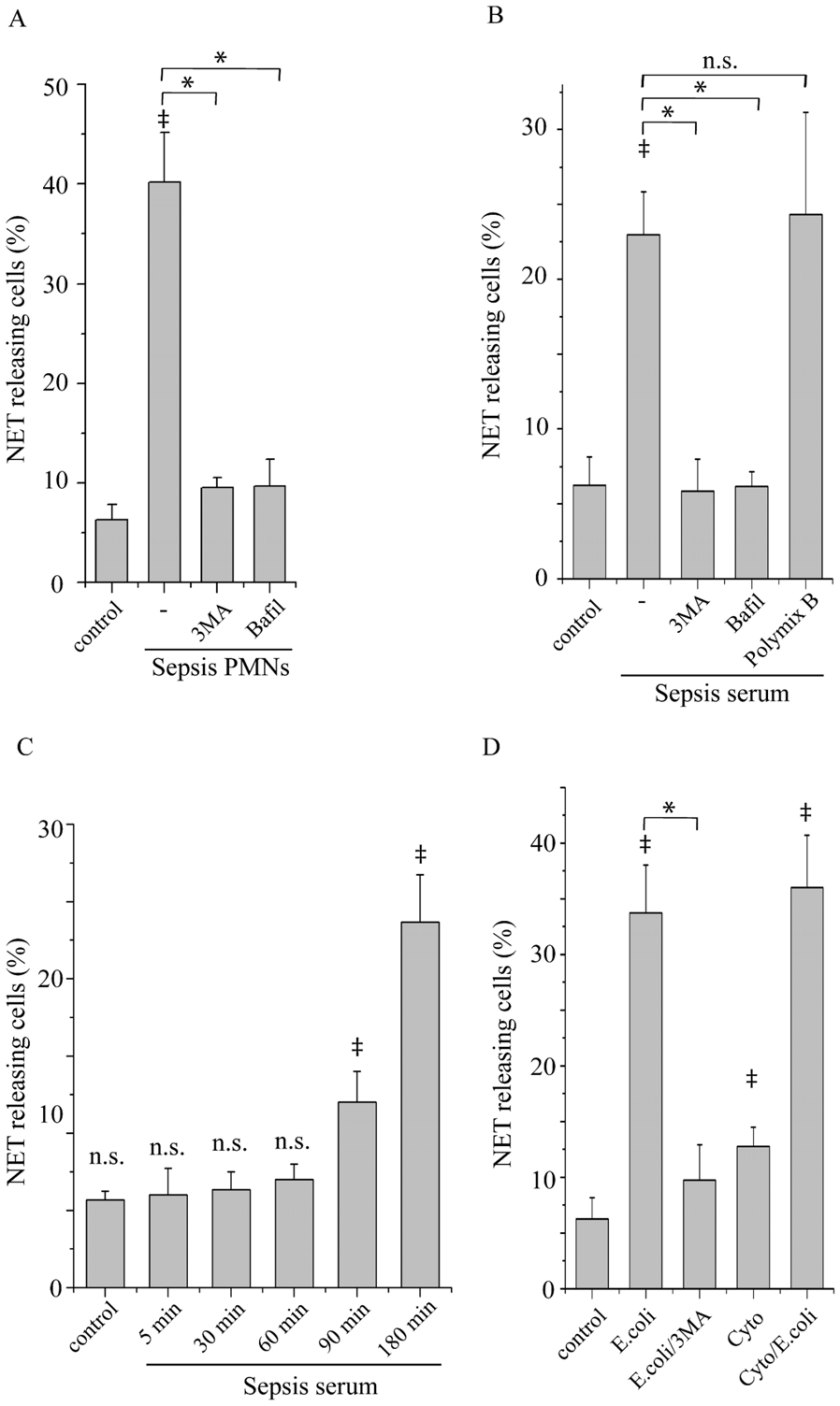
NET formation by sepsis neutrophils and control neutrophils under inflammatory stimuli. Percentage of neutrophils releasing NETs from (A) sepsis patients (Sepsis PMNs) and (B) neutrophils from control subjects treated with sepsis serum (Sepsis serum) or (D) phagocytosing opsonized *E. coli* bacteria (E. coli) alone or in the presence of a mixture of TNF-α, IL-1β and G-CSF (Cyto/E.coli) after 3 h or incubation in the presence of 3-MA (3MA) or bafilomycin A1 (Bafil) or polymixin B (B) or not. The percentage of NET releasing neutrophils after treatment with sepsis serum at different time points is shown in (C). Untreated neutrophils from healthy subjects were used as control. Quantification of percentage was performed on the count of 200 cells per sample. Data are representative of six independent experiments (A, B, D) or three independent experiments (C) and presented as mean ± SD. (‡*P*<.05 compared to control, *P<.05).

The previously described implication of autophagy in NET release [20], [21] prompted us to investigate whether autophagy inhibition attenuates the release of NETs from neutrophils derived from patients hospitalized with gram-negative sepsis (sepsis neutrophils). These cells released NETs after 3 h of incubation, as demonstrated by co-staining with myeloperoxidase (MPO) and DAPI (Fig. 1A, Fig. S1). Notably, treatment of sepsis neutrophils with 3 methyl-adenine (3-MA), a modulator of class III PI3Ks that inhibits autophagy, or bafilomycin A1, which impairs the formation of acidified autophagosomes, abrogated NET release (Fig. 1A, Fig. S1). Moreover, incubation of neutrophils from control subjects (control neutrophils) with serum from patients with sepsis resulted in NET release in a time-dependent manner (Fig. 1B–C, Fig. S1), which was also inhibited by 3-MA or bafilomycin A1 (Fig. 1B). Treatment of sepsis serum with an LPS inhibitor (Polymyxin B) did not affected the percentage of NET releasing cells (Fig. 1B), suggesting that the presence of LPS in serum from patients with sepsis was not critical for NET formation.

### TF is Localized in NETs Released by Sepsis Neutrophils

**Figure 2:**
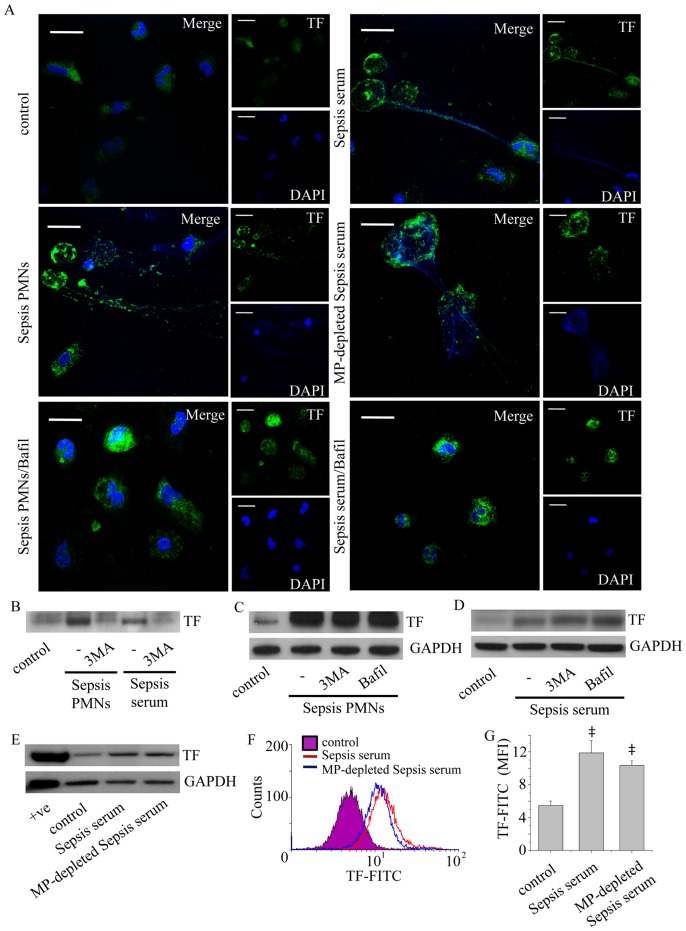
Identification of TF in NETs released by sepsis neutrophils. (A) Neutrophils from patients with sepsis (Sepsis PMNs) and control neutrophils treated with sepsis serum (Sepsis serum) or microparticle-depleted sepsis serum (MP-depleted Sepsis serum) were treated for 3 h and the localization of TF on NETs was assessed by confocal microscopy (z stack analysis, 0.3 µm per plane). Treatment with bafilomycin A1 (Bafil) inhibited the release of these structures. One representative out of four independent experiments is shown (DNA labeled with DAPI; blue, anti-TF monoclonal antibody; green) (original magnification 600×). Scale bar represents 10 µΜ. (B) TF levels in proteins isolated from NETs released by sepsis neutrophils or control neutrophils treated with sepsis serum, assessed by immunoblotting. The inhibitory effect of 3-MA in NET release and subsequent presence of TF in NETs is shown. (C) 3-MA and bafilomycin A1 did not affect TF expression in cell lysates from sepsis PMNs incubated for 1 h and (D) in control neutrophils treated with sepsis serum for the same period of time. One out of four independent experiments is shown in B–D. (E–F) TF levels in control neutrophils treated with sepsis serum or microparticle-depleted sepsis serum as demonstrated by western blotting (E) and flow cytometry analysis (F–G). One out of three independent experiments is shown in E–F. Data in (G) are presented as mean ± SD. (‡*P*<.05 compared to control).

The critical role of blood-born TF in the pathogenesis of sepsis [1], [2] motivated the investigation of TF presence in NETs formed by sepsis neutrophils. TF was identified in NETs released from these cells (Fig. 2A–B) and control neutrophils treated with sepsis serum (Fig. 2A–B), as assessed by confocal microscopy and immunoblotting of NET-derived proteins. As expected, 3-MA (Fig. 2B) or bafilomycin A1 (Fig. 2A) attenuated NET formation and the consequent TF externalization.

To further investigate the possible effect of 3-MA or bafilomycin A1 on intracellular TF levels, sepsis neutrophils or control neutrophils treated with sepsis serum were incubated in the presence of 3-MA or bafilomycin A1 for 1 h. This incubation period was selected to avoid the possible implication of TF externalization through NETs in the quantification of TF levels. TF expression levels in sepsis neutrophils (Fig. 2C) and control neutrophils treated with sepsis serum (Fig. 2D) were not altered in the presence of 3-MA or bafilomycin A1, suggesting that these agents do not interfere with the expression of TF in neutrophils.

To address whether *de novo* TF production contributes to enhanced TF expression in neutrophils, microparticle-depleted sepsis serum was acquired by centrifugation (Fig. S2). Treatment with microparticle-depleted sepsis serum still induced TF expression, as observed by immunofluorescence microscopy (Fig. 2A), immunoblotting (Fig. 2E) and flow cytometry analysis (Fig. 2F–G).

### Inflammatory Mediators Involved in Sepsis Induce the Release of TF-bearing NETs

**Figure 3:**
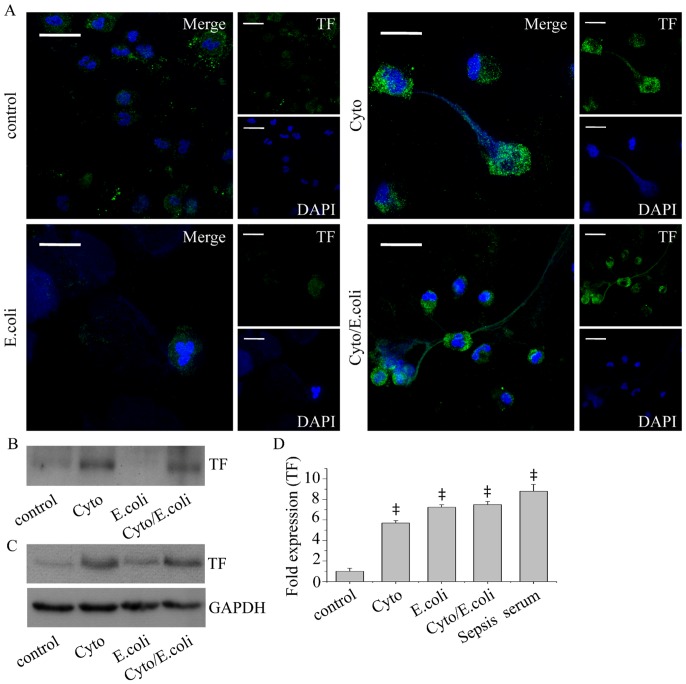
TF localization in NETs after *in vitro* stimulations. (A) Detection of TF in NETs released by control neutrophils treated with TNF-α, IL-1β and G-CSF (Cyto), or *E. coli* alone (E. coli) or *E. coli* in the presence of the aforementioned cytokines (Cyto/E. coli), as assessed by confocal microscopy (z stack analysis, 0.3 µm per plane). One representative out of four independent experiments is shown (DNA labeled with DAPI; blue, anti-TF monoclonal antibody; green) (original magnification 600×). Scale bar represents 10 µΜ. (B) TF levels in NET-isolated proteins and (C) cell lysates, assessed by immunoblotting. One representative out of four independent experiments is shown. (D) *TF* mRNA levels in untreated control neutrophils (control) or treated with TNF-α, IL-1β and G-CSF (Cyto) or *E. coli* alone (E. coli) or in the presence of the aforementioned cytokines (Cyto/E. coli) or serum from patients with sepsis (Sepsis serum). Data are representative of four independent experiments and presented as mean ± SD. (‡*P*<.05).

To simulate septic conditions, normal neutrophils were treated with opsonized *E. coli* for 3 h. We observed NET formation from *E. coli* phagocytosing neutrophils, while the addition of 3-MA or bafilomycin A1 30 min after the initiation of phagocytosis completely inhibited NET release (Fig. 1D, Fig. S1). We next studied the localization of TF in NETs produced after bacterial phagocytosis. Confocal microscopy (Fig. 3A) and immunoblotting of NET-derived proteins did not demonstrate TF presence in NETs released by *E. coli* phagocytosing neutrophils (Fig. 3A–B). Moreover, immunoblotting did not show elevated TF protein levels in these cell lysates (Fig. 3C). To further simulate septic conditions and considering the reported implication of TNF-α and IL-1β in the pathomechanism of sepsis [31], control neutrophils were stimulated with TNF-α, IL-1β and G-CSF. Even though the percentage of NET-releasing cells was lower compared to *E. coli* phagocytosing neutrophils (Fig. 1C), we observed TF localization in NETs (Fig. 3A–B), while cellular TF levels were upregulated (Fig. 3C). To further enhance NET production, neutrophils were treated with the aforementioned factors in the presence of *E. coli* bacteria. We detected increased release of NETs decorated with TF (Fig. 3A–B), while cellular TF protein levels were elevated (Fig. 3C). Notably, *TF* mRNA levels were also found elevated in neutrophils incubated with all the aforementioned inflammatory stimuli (Fig. 3D), even with *E. coli* alone, suggesting a role for the cytokine mixture in the TF post-transcriptional regulation.

### Thrombin Generation and PAR Signaling are Triggered by TF-bearing NETs

**Figure 4:**
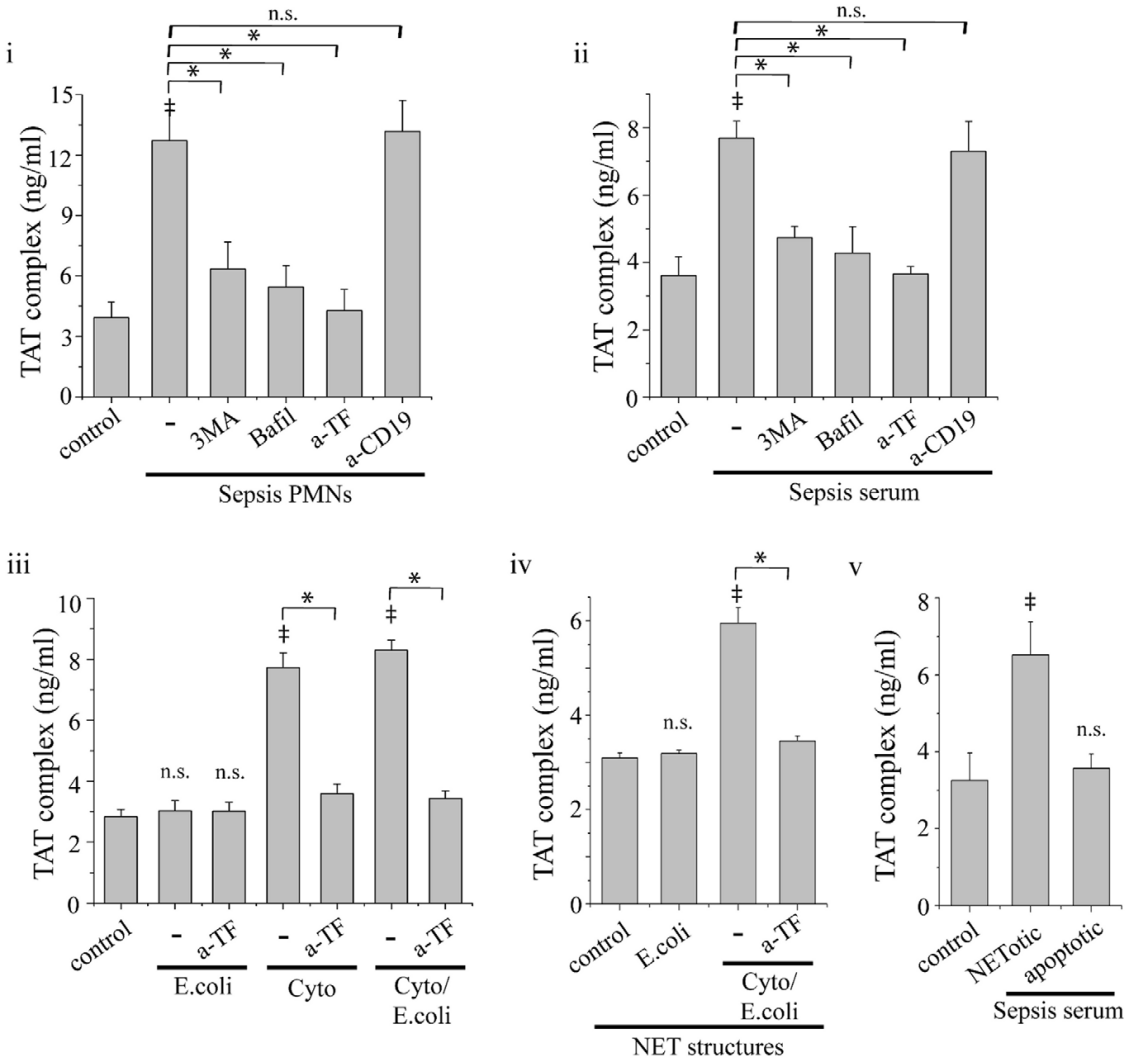
Thrombin generation by NET-forming neutrophils. Thrombin-antithrombin (TAT) complex levels in culture supernatants from sepsis neutrophils (i) or control neutrophils treated with sepsis serum (ii). Treatment with 3-MA, bafilomycin A1 or anti-TF mAb (a-TF) inhibited this effect. (iii) TAT complex levels in culture supernatants from control neutrophils treated with *E. coli* (E.coli), or TNF-α, IL-1β and G-CSF alone (Cyto) or primed with cytokines before the addition of *E. coli* (Cyto/E. coli) and the respective inhibition studies using anti-TF mAb. (iv) Induction of TAT complex levels by isolated NET structures released by neutrophils treated as in (iii). (v) TAT complex levels in culture supernatants from control neutrophils treated with sepsis serum and subsequently irradiated to undergo apoptosis, or not. Control neutrophils treated with normal serum (control) and anti-CD19 mAb (a-CD19) served as control. Data are representative of four independent experiments and presented as mean ± SD. (*P<.05, ‡*P*<.05 compared to control, ns  =  non significant).

We next assessed whether the externalization of TF in NETs is involved in the activation of coagulation system by sepsis neutrophils. TAT complex levels were increased in culture supernatants from sepsis neutrophils (Fig. 4i) and control neutrophils treated with sepsis serum (Fig. 4ii) or TNF-α, IL-1β and G-CSF (Fig. 4iii). TF blockade with anti-TF mAb abolished the aforementioned increase of TAT complex levels (Fig. 4i–iii), while it had no effect on the production of NETs (data not shown). On the other hand, *E*. *coli* phagocytosis alone was not sufficient for thrombin generation, despite the formation of NETs (Fig. 4iii). Moreover, treatment with 3-MA or bafilomycin A1 normalized TAT levels in culture supernatants from sepsis neutrophils or control neutrophils treated with sepsis serum or the aforementioned inflammatory agents (Fig. 4i–iii). Additionally, incubation of control serum with isolated NET structures demonstrated similar results regarding thrombin generation. NET structures from control neutrophils treated with cytokines and *E.coli* induced TAT complex levels, while NETs from neutrophil treated only with *E.coli* did not (Fig. 4iv). This ability was also TF dependent, as suggested by the inhibitory effect of anti-TF neutralizing antibodies (Fig. 4iv).

**Figure 5:**
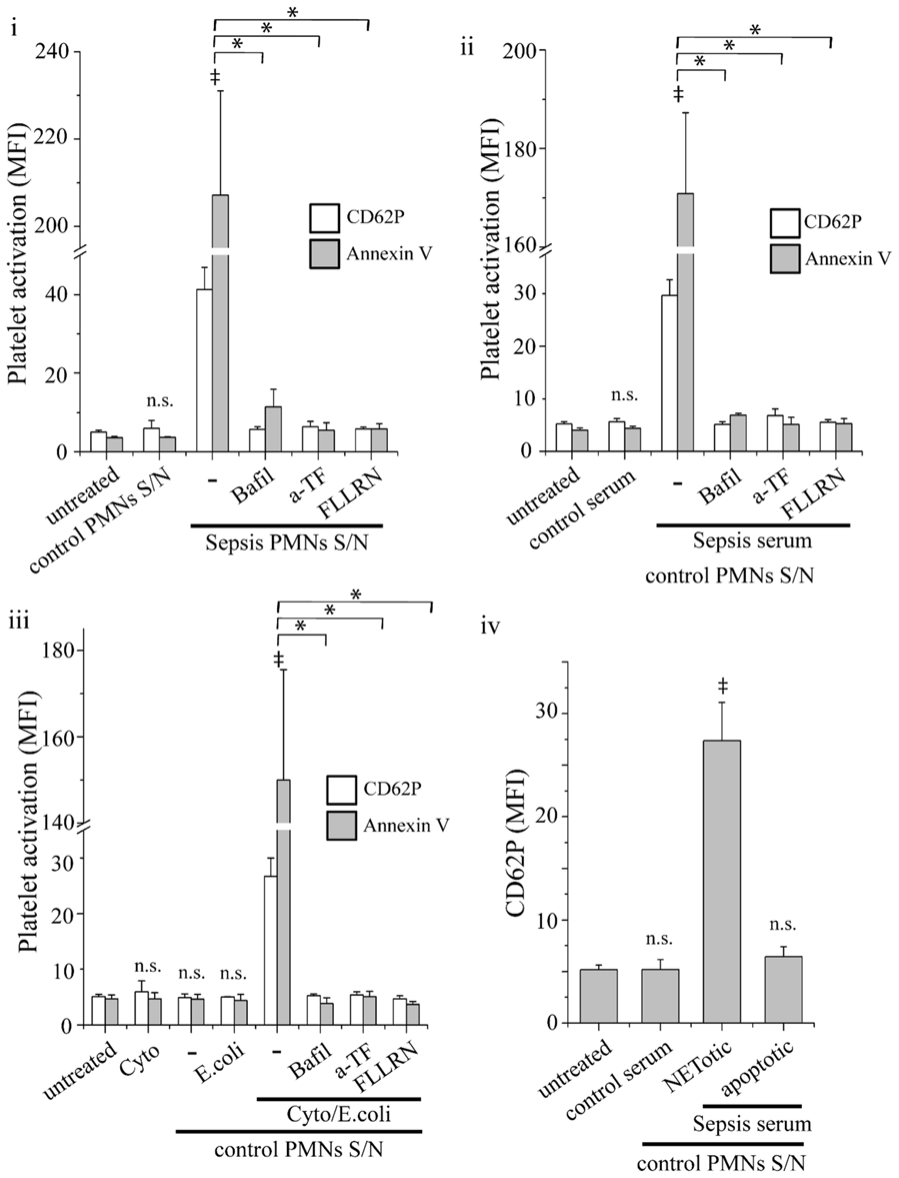
Platelet activation by NET-forming neutrophils. Expression of CD62P (white bar) and annexin V (grey bar) in (i) sepsis neutrophil, (ii) control neutrophils treated with sepsis serum or (iii) treated with *E. coli* (E.coli), or TNF-α, IL-1β and G-CSF alone (Cyto) or primed with cytokines before the addition of *E. coli* (Cyto/E. coli). Treatment with bafilomycin A1 or anti-TF mAb in neutrophil cultures or inhibition of PAR-1 signaling in platelets with FLLRN inhibited this effect. (iv) CD62P expression in control platelets stimulated with supernatants (S/N) from control neutrophils treated with sepsis serum and subsequently irradiated to undergo apoptosis, or not. Untreated platelets from control subjects (untreated), or incubated with cytokines (Cyto) or with supernatants from control neutrophils treated with normal serum were used as controls. Expression of CD62P and annexin V was assessed by flow cytometry and presented as mean fluorescent intensity (MFI). Data are representative of four independent experiments and presented as mean ± SD. (*P<.05, ‡*P*<.05 compared to control, ns  =  non significant).

Platelet activation by thrombin was further investigated, in order to study its ability for signaling through PAR-1. Platelets treated with culture supernatant from sepsis neutrophils (Fig. 5i) or normal neutrophils incubated with sepsis serum (Fig. 5ii) or the aforementioned cytokines (Fig. 5iii) expressed increased levels of CD62P and annexin V, as assessed by flow cytometry. Inhibition of NET release with bafilomycin A1 abolished this effect (Fig. 5i–iii). TF blockade in neutrophil cultures and PAR-1 inhibition with FLLRN in culture supernatants also inhibited CD62P and annexin V expression in platelets (Fig. 5i–iii). Moreover, culture supernatant from *E. coli* phagocytosing neutrophils had no effect in the expression of these markers in platelets (Fig. 5iii).

To compare NET-forming neutrophils with apoptotic ones in their ability to induce thrombin generation, control neutrophils were incubated with sepsis serum for 1 h, washed and incubated for 2 h in normal serum. To induce apoptosis, cells were irradiated after being treated with sepsis serum. TAT levels in culture supernatants from irradiated cells were significantly lower compared with those from non-irradiated cells treated with sepsis serum and were comparable to neutrophils treated with normal serum (Fig. 4v). Moreover, culture supernatants from apoptotic cells did not induce significant elevation of CD62P levels in platelets (Fig. 5iv).

### Localization of TF in LC3 Positive Structures

**Figure 6:**
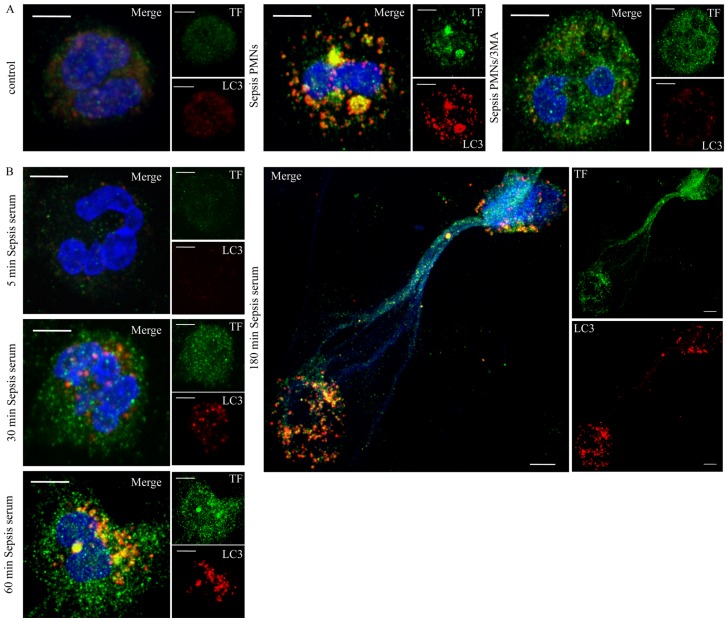
Localization of TF in LC3B positive structures in sepsis neutrophils and control neutrophils treated with sepsis serum. (A) Sepsis neutrophils were incubated for 1 h and the intracellular distribution of TF and LC3B was assessed by confocal microscopy (z stack analysis, 0.3 µm per plane). Formation of LC3B positive punctuated structures in sepsis neutrophils (Sepsis PMNs) and colocalization of TF with LC3B. Treatment with 3-MA (Sepsis PMNs/3MA) inhibited the formation of LC3B positive structures and resulted in a disperse TF staining. (B) TF and LC3B localization in control neutrophils treated with sepsis serum at various time points. (DNA labeled with DAPI; blue, anti-TF mAb; green, anti-LC3B mAb; red) (original magnification 1000×). One out of three independent experiments is shown in A–B. Scale bar represents 5 µM in A–B.

To further assess whether autophagy is involved in the delivery of intracellular TF to NETs, the localization of TF and the LC3B was scrutinized in sepsis neutrophils. After 1 h of incubation, we observed formation of TF aggregates, which were colocalized with LC3B-coated structures (Fig. 6A, Fig. S5), as assessed by confocal microscopy. Inhibition of autophagy by 3-MA attenuated the aggregation of LC3B and resulted in a more disperse TF staining (Fig. 6A). Additionally, control neutrophils were treated with sepsis serum for different time points and the localization of TF and LC3B were studied. We detected minimal TF levels after 5 min of stimulation, which were up-regulated at 30 min. TF colocalization with LC3B-positive structures was observed at 1 h of stimulation (Fig. 6B, Fig. S3A) and NETs decorated with TF were detected after 3 h (Fig. 6B). Of interest, TF colocalization with LC3B was observed in the cytoplasm, and partially in NETs, of NET releasing cells (Fig. 6B). The same phenomenon was also observed in control neutrophils simultaneously treated with inflammatory mediators and *E. coli* (Fig. S3B). As expected, neutrophils treated with *E. coli* alone exhibited increased formation of LC3B positive structures, while TF levels remained unaltered (Fig. S3B).

**Figure 7:**
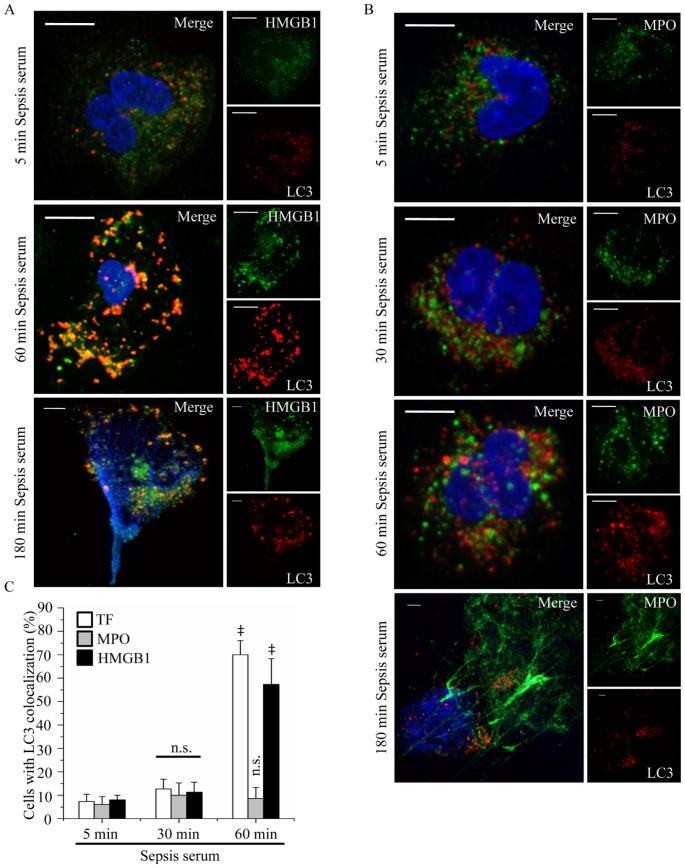
HMGB1, but not MPO, is colocalized with LC3B in control neutrophils treated with sepsis serum. Control neutrophils were treated at different time points with sepsis serum and the colocalization of HMGB1 (A) and MPO (B) with LC3B-coated structures was assessed by confocal microscopy (z stack analysis, 0.3 µm per plane). (DNA labeled with DAPI; blue, anti-LC3B mAb; red, anti-HMGB1; green in A, anti-MPO; green in B) (original magnification 1000×). One out of three independent experiments is shown in A–B. Scale bar represents 5 µM. (C) Percentage of neutrophils indicating colocalization of LC3B with TF, HMGB1 or MPO. Quantification of percentage was performed on the count of 50 cells per sample. Data are representative of three independent experiments and presented as mean ± SD. (‡ *P*<.05 compared to control, *P<.05).

Next, we studied whether the engulfment of proteins localized in NETs in LC3B-coated endosomes is a generalized phenomenon for NET targeting. Colocalization of high mobility group box-1 (HMGB1) with LC3B-positive structures was observed in neutrophils treated with sepsis serum following the same pattern with TF (Fig. 7A, C). On the other hand, we did not detect any colocalization of MPO with LC3B positive structures under these conditions (Fig. 7B–C), suggesting that MPO, which is located in the primary granules, does not follow the same route to reach the NETs.

**Figure 8:**
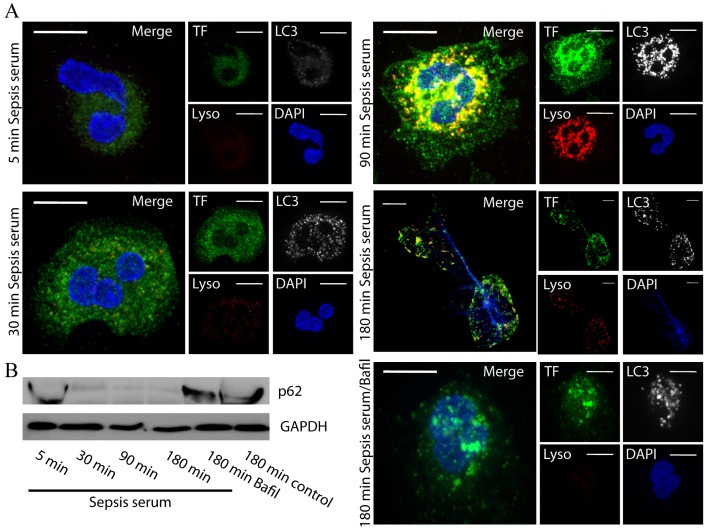
Formation of autophagolysosomes in control neutrophils treated with sepsis serum and degradation of p62/SQSTM1. A. Control neutrophils were treated at different time points with sepsis serum and the localization of TF in autophagolysosomes was assessed. Autophagolysosomes were visualized as LC3B and LysoTracker double positive structures and were assessed by confocal microscopy (z stack analysis, 0.3 µm per plane). (DNA labeled with DAPI; blue, anti-LC3B mAb; white, anti-TF; green, LysoTracker; red) (original magnification 1000×). Scale bar represents 5 µM. B. p62/SQSTM1 protein levels in control neutrophils treated with sepsis serum at different time points. Treatment with bafilomycin A1 inhibited p62/SQSTM1 degradation. One out of three independent experiments is shown in A–B.

Moreover, we investigated whether the observed TF and HMGB1 containing autophagosomes matured to autophagolysosomes. The LC3-coated structures were stained positive with LysoTracker, suggesting the formation of acidified autophagosomes (Fig. 8A, Fig. S4A, Fig. S5). As expected, incubation with bafilomycin A1 attenuated the acidification of autophagosomes (Fig. 8A).

To verify the induction of autophagic machinery in our model, p62/SQSTM1 immunoblotting was utilized. We observed degradation of p62/SQSTM1 in control neutrophils treated with sepsis serum in a time dependent manner, while inhibition of autophagolysosomal fusion by bafilomycin A1 abolished this phenomenon (Fig. 8B).

## Discussion

In the present study, we provide evidence for the release of TF through NETs by neutrophils isolated from patients with sepsis. Moreover, it is demonstrated that an autophagy-based mechanism is implicated in the extracellular localization of TF in NETs. This form of TF induces *in vitro* thrombin generation and subsequent PAR-1 signaling, suggesting a possible involvement in coagulopathy and inflammatory responses that characterize sepsis.

The activation of coagulation is a critical step in the continuum of events associated with sepsis. It participates *via* fibrin deposition in the prevention of microbial dissemination [32], [33]. However, the activation of coagulation and the formation of thrombi in the microvasculature may also lead to disseminated intravascular coagulation (DIC), resulting in multi-organ failure [34], [35]. Studies in both animal and human models suggest the implication of circulating TF in the progression towards DIC [36], [37]. Except from increased thrombogenicity, TF/thrombin/PARs pathway participates in the induction and maintenance of systemic inflammatory response during sepsis [38]–[40].

Previous studies implicated neutrophils in thrombogenicity. Neutrophil depletion attenuated thrombus formation in a mouse model of deep vein thrombosis [41] and plaque formation in atherosclerosis [42]. Even though *de novo* TF production [43]–[45] or acquisition of microparticle-derived TF [46] is still a matter of debate, *ex vivo* and *in vivo* studies demonstrated that TF in neutrophils plays a significant role in the induction of both coagulation and inflammation [43]–[45]. However, the contribution of neutrophils in circulating TF levels, responsible for thrombotic or non-thrombotic disease manifestations, has not been completely elucidated.

In this study, we describe a novel role for neutrophils in TF-dependent thrombogenicity. Even though the acquisition of microparticle-derived TF by neutrophils has been previously demonstrated [46], in this *ex vivo* model the inflammatory milieu of sepsis induced *de novo* TF production by neutrophils, as indicated by the effect of microparticle-depleted serum. Moreover, neutrophils treated with inflammatory mediators also expressed TF in mRNA and protein levels. However, *E. coli* phagocytosis in the absence of inflammatory mediators was not sufficient for TF production, despite the observed upregulation of *TF* mRNA levels. This finding is in accordance with the previously reported inability of lipopolysaccharide-treated granulocytes to produce TF [46].

The extracellular delivery of TF in NETs resulted in thrombin generation in culture supernatants and subsequent signaling through PAR-1. The key role of TF in NET-dependent activation of coagulation system was indicated by the inhibitory effect of anti-TF mAb. This was also supported by the lack of thrombin generation in culture supernatants from *E. coli* phagocytosing neutrophils, which released NETs that were not decorated with TF. Additionally, the generation of thrombin by isolated NETs demonstrates that TF localized in NETs is functional. The ability of NET-forming neutrophils to generate thrombin was further juxtaposed with that of apoptotic cells. Induction of apoptosis in neutrophils treated with sepsis serum prevented thrombin generation in culture supernatants. These findings propose that the extracellular delivery of TF through NETs plays a key role in the activation of coagulation system.

The aforementioned contribution of NET generation to thrombus formation has been previously studied. It has been shown that platelet-neutrophil interaction in the microvasculature results not only in NET formation but also in the obstruction of blood flow and tissue damage [13]. Recent studies demonstrated a role for NET formation in sepsis-associated vasculopathy, due to platelet entrapment and activation [14]. Of interest, during the preparation of this article, a role for NETs in thrombus formation due to FXII activation has been demonstrated in a mouse model of deep vein thrombosis [41]. In this mouse model, TF was observed in NETs, which is in consistency with our study.

We also observed the implication of an autophagy-related pathway in both NET release and protein trafficking on NETs. PI3K signaling and endosomal acidification inhibition abrogated NET release, suggesting the involvement of autophagy, which is in accordance with previous reports [20], [21]. We also demonstrated the involvement of an autophagy-related mechanism in the delivery of TF in NETs. TF was accumulated in LC3B-coated vacuoles and then externalized and localized in NETs. These vacuoles were also identified as acidified autophagosomes by staining with LysoTracker. This pathway also participated in the delivery of HMGB1 to NETs. This nuclear protein, which is implicated in the pathogenesis of sepsis [47], is translocated to the cytosol and released through NETs upon neutrophil activation [21], [48]. On the other hand, MPO, a granule protein, did not use this particular pathway.

These findings suggest that the inclusion of certain proteins into LC3B-coated endosomes, either integral membrane proteins, like TF, or cytosolic, such as HMGB1, facilitates their delivery to NETs. Since no detectable degradation is observed inside the autophagosomes, at least for the time-scale of our experiments, a key role for an autophagy-based pathway is proposed as an unconventional trafficking pathway to NETs. However, proteins expressed in primary granules, such as MPO, could follow alternative routes to NETs. An autophagy-based pathway for TF delivery to NETs is in accordance with recent studies demonstrating autophagy-based pathways being involved in the secretion of cytosolic proteins. It has been recently reported that the release of acyl-CoA-binding protein by murine astrocytes [49] and IL-1β and HMGB1 by bone marrow derived macrophages is based on an autophagy-dependent secretory pathway through LC3B positive organelles [50].

In conclusion, our data, in conjunction with previous studies, provide novel insight into the implication of neutrophil derived TF in human sepsis thrombogenicity, describing a novel form of circulating TF. Activated neutrophils, expressing high amounts of TF, are recruited in vast numbers in the vasculature during sepsis. The release of TF-bearing NETs at the sites of inflammation may result in the localized activation of coagulation cascade, leading to microangiopathy and activation of several cell populations, including platelets and endothelial cells, through PAR signaling. Moreover, an autophagy-related pathway emerges as a critical component in neutrophil function during sepsis by regulating the translocation of certain neutrophil proteins to NETs, including TF and HMGB1. Targeting NET formation and/or protein trafficking through autophagy could emerge as a potential strategy in the treatment of coagulopathy that characterizes sepsis.

## Supporting Information

Figure S1 **NET formation by septic neutrophils and control neutrophils treated with inflammatory stimuli.** NET release by untreated neutrophils from patients with sepsis (Septic PMNs) and control neutrophils treated with septic serum (Septic serum) or phagocytosing opsonized *E. coli* bacteria (E. coli) alone or in the presence of a mixture of TNF-α, IL-1β and G-CSF (Cyto/E.coli) after 3 h incubation, assessed by immunofluorescence microscopy. Inhibition of NET formation in neutrophils treated with 3-MA (3MA) or bafilomycin A1 (Bafil). Untreated neutrophils from healthy subjects were used as control. One representative out of six independent experiments is shown (DNA labeled with DAPI; blue, anti-MPO monoclonal antibody; green) (original magnification 400×). Scale bar represents 30 µM.
(TIF)

Figure S2 **Microparticle depletion from sepsis serum.** FACS analysis of sepsis serum and microparticle depleted sepsis serum by centrifugation as indicated by Annexin V-FITC staining. Isotype: sepsis serum stained with mouse IgG1-FITC. One out of three independent experiments is shown.
(TIF)

Figure S3 **Localization of TF or MPO in LC3B-coated structures in control neutrophils treated with inflammatory stimuli.** (**A**) Single plane analysis of TF or MPO localization in LC3B-coated structures in control neutrophils treated with septic serum for 60 mins. (DNA labeled with DAPI; blue, anti-TF monoclonal antibody or anti-MPO monoclonal antibody; green, anti-LC3B mAb; red) (original magnification 1000×). Arrows indicate LC3-coated autophagosomes. Scale bar represents 5 µM. (**B**) TF and LC3B localization in control neutrophils treated with inflammatory cytokines and/or *E. coli* at various time points. Colocalization of TF with LC3B is detected both intracellularly and in NETs only in neutrophils treated with both cytokines and *E. coli*. Neutrophils treated with *E. coli* alone do not express TF. (DNA labeled with DAPI; blue, anti-TF mAb; green, anti-LC3B mAb; red) (original magnification 1000×). One out of three independent experiments is shown. Scale bar represents 5 µM.
(TIF)

Figure S4 **Localization of HMGB1 or MPO in late autophagosomes in control neutrophils treated septic serum.** Control neutrophils were treated at different time points with septic serum and the colocalization of HMGB1 (A) and MPO (B) with autophagolysosomes was assessed. Autophagolysosomes were visualized by confocal microscopy as LC3B and LysoTracker double positive structures (z stack analysis, 0.3 µm per plane). (DNA labeled with DAPI; blue, anti-LC3B mAb; white, anti-MPO or anti-HMGB1 mAB; green, LysoTracker; red) (original magnification 1000×). One out of three independent experiments is shown. Scale bar represents 5 µM.
(TIF)

Figure S5 **Comparison of z-stack with single plane analysis in colocalization studies.** z-axies and Single Plane analysis of TF and LC3 in sepsis PMNs or control PMNs treated with sepsis serum. Scale bar represents 5 µM.
(TIF)
