# Supplementary figures and images for: Autophagy Mediates the Delivery of Thrombogenic Tissue Factor to Neutrophil Extracellular Traps in Human Sepsis
Source: PLoS One. 2012 Sep 19;7(9):e45427. doi: 10.1371/journal.pone.0045427 (PMC3446899; doi:10.1371/journal.pone.0045427)

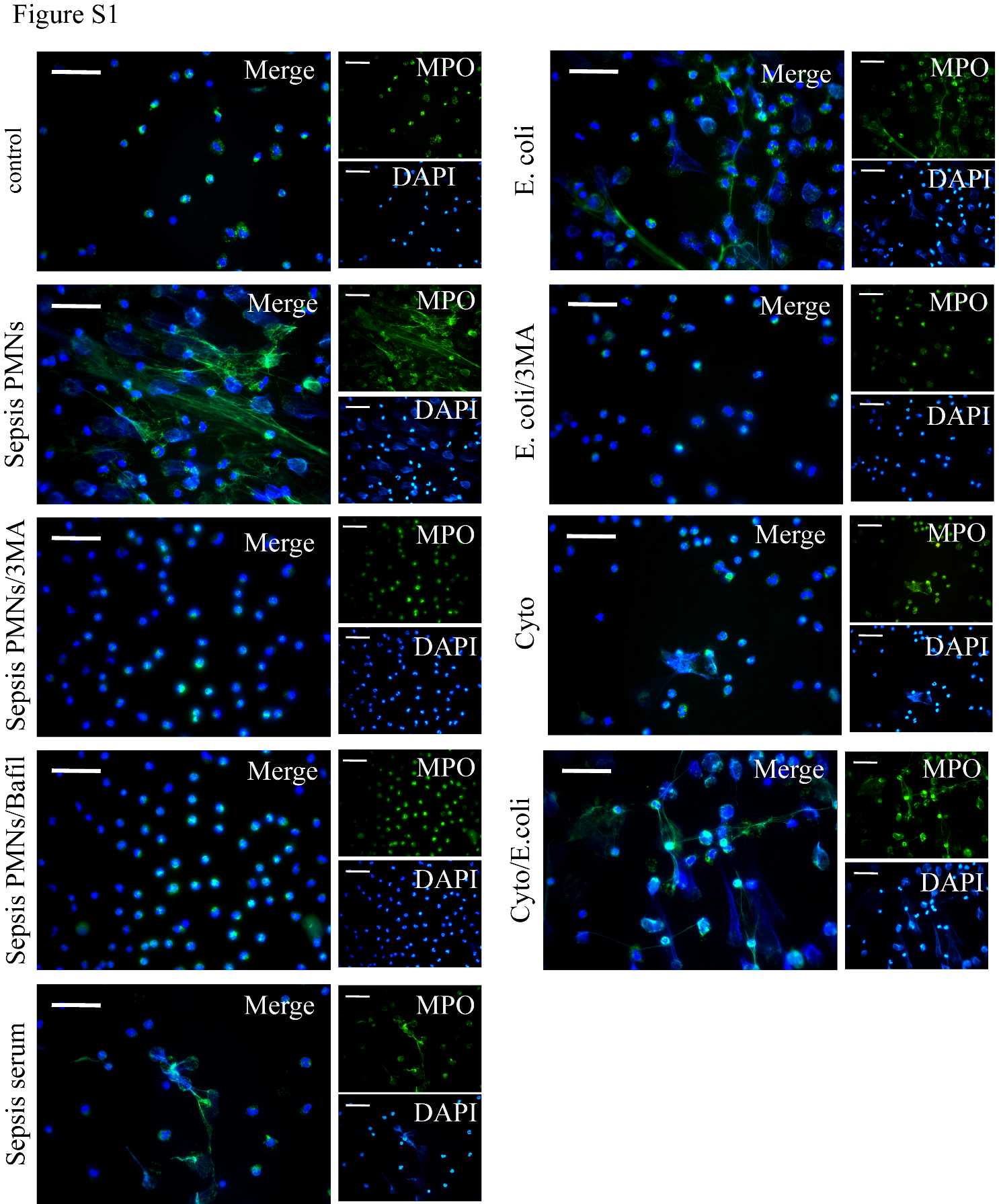

Supplement: Figure S1 — NET formation by septic neutrophils and control neutrophils treated with inflammatory stimuli. NET release by untreated neutrophils from patients with sepsis (Septic PMNs) and control neutrophils treated with septic serum (Septic serum) or phagocytosing opsonized E. coli bacteria (E. coli) alone or in the presence of a mixture of TNF-α, IL-1β and G-CSF (Cyto/E.coli) after 3 h incubation, assessed by immunofluorescence microscopy. Inhibition of NET formation in neutrophils treated with 3-MA (3MA) or bafilomycin A1 (Bafil). Untreated neutrophils from healthy subjects were used as control. One representative out of six independent experiments is shown (DNA labeled with DAPI; blue, anti-MPO monoclonal antibody; green) (original magnification 400×). Scale bar represents 30 µM. (TIF) [file pone.0045427.s001.tif]

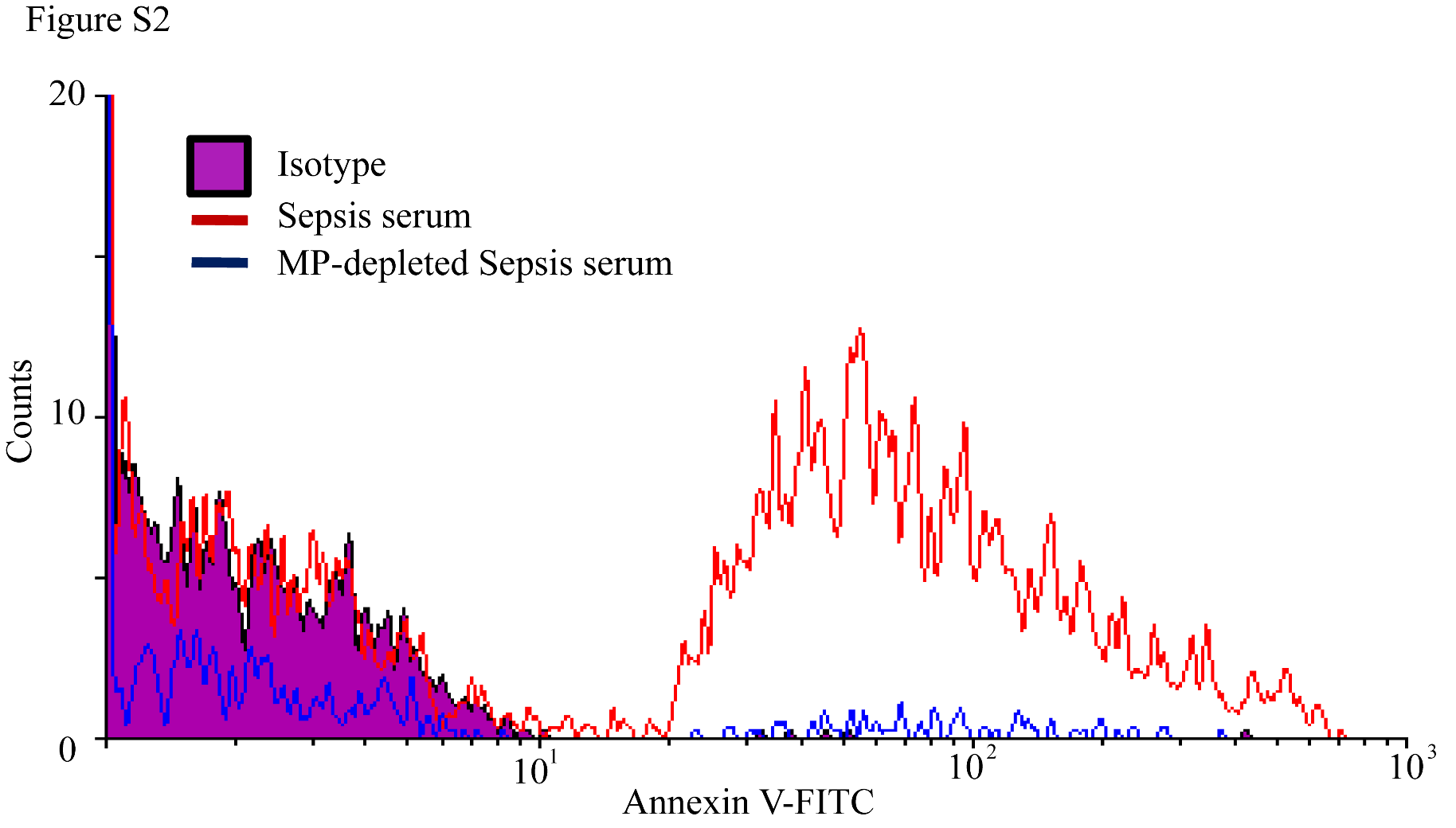

Supplement: Figure S2 — Microparticle depletion from sepsis serum. FACS analysis of sepsis serum and microparticle depleted sepsis serum by centrifugation as indicated by Annexin V-FITC staining. Isotype: sepsis serum stained with mouse IgG1-FITC. One out of three independent experiments is shown. (TIF) [file pone.0045427.s002.tif]

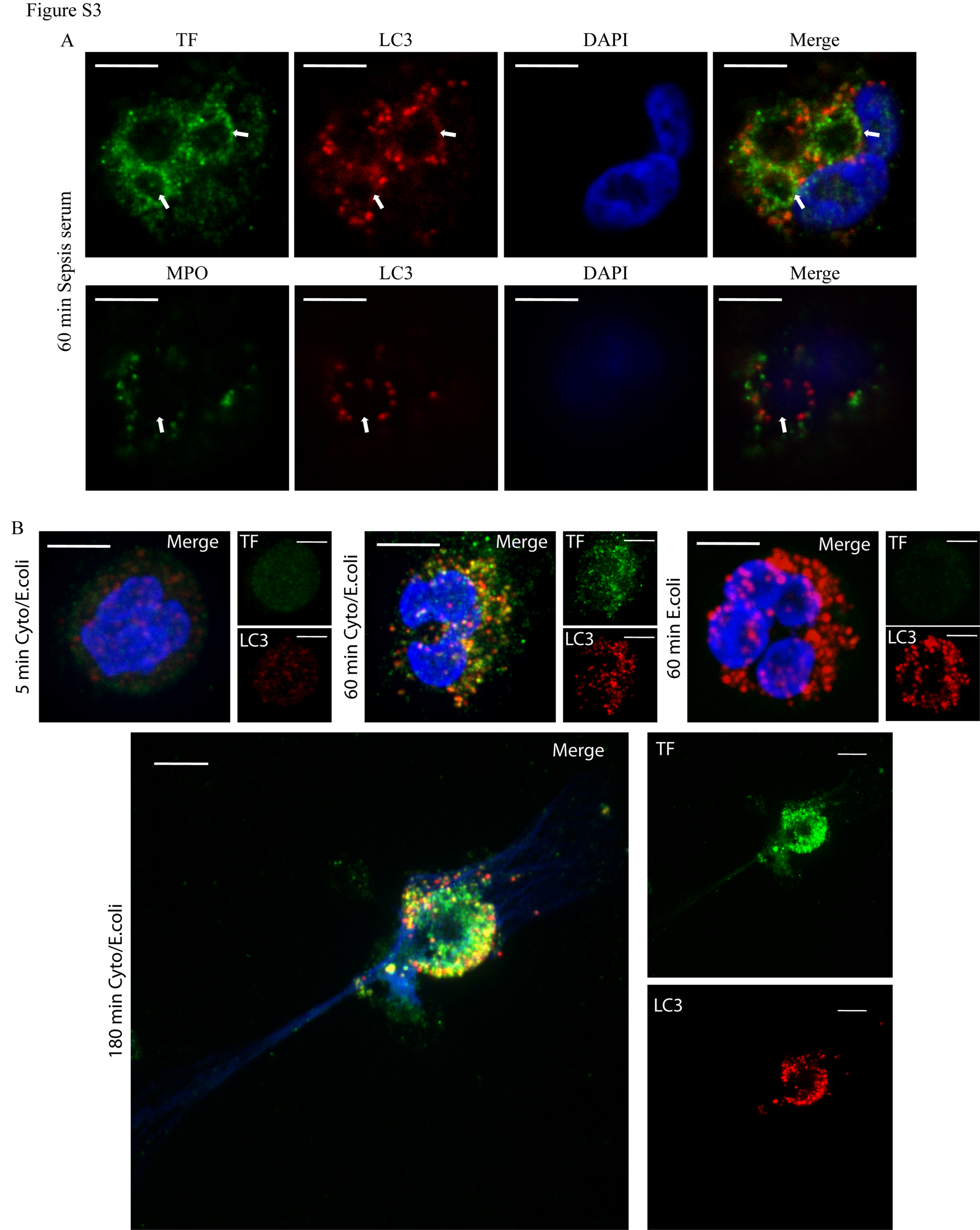

Supplement: Figure S3 — Localization of TF or MPO in LC3B-coated structures in control neutrophils treated with inflammatory stimuli. (A) Single plane analysis of TF or MPO localization in LC3B-coated structures in control neutrophils treated with septic serum for 60 mins. (DNA labeled with DAPI; blue, anti-TF monoclonal antibody or anti-MPO monoclonal antibody; green, anti-LC3B mAb; red) (original magnification 1000×). Arrows indicate LC3-coated autophagosomes. Scale bar represents 5 µM. (B) TF and LC3B localization in control neutrophils treated with inflammatory cytokines and/or E. coli at various time points. Colocalization of TF with LC3B is detected both intracellularly and in NETs only in neutrophils treated with both cytokines and E. coli. Neutrophils treated with E. coli alone do not express TF. (DNA labeled with DAPI; blue, anti-TF mAb; green, anti-LC3B mAb; red) (original magnification 1000×). One out of three independent experiments is shown. Scale bar represents 5 µM. (TIF) [file pone.0045427.s003.tif]

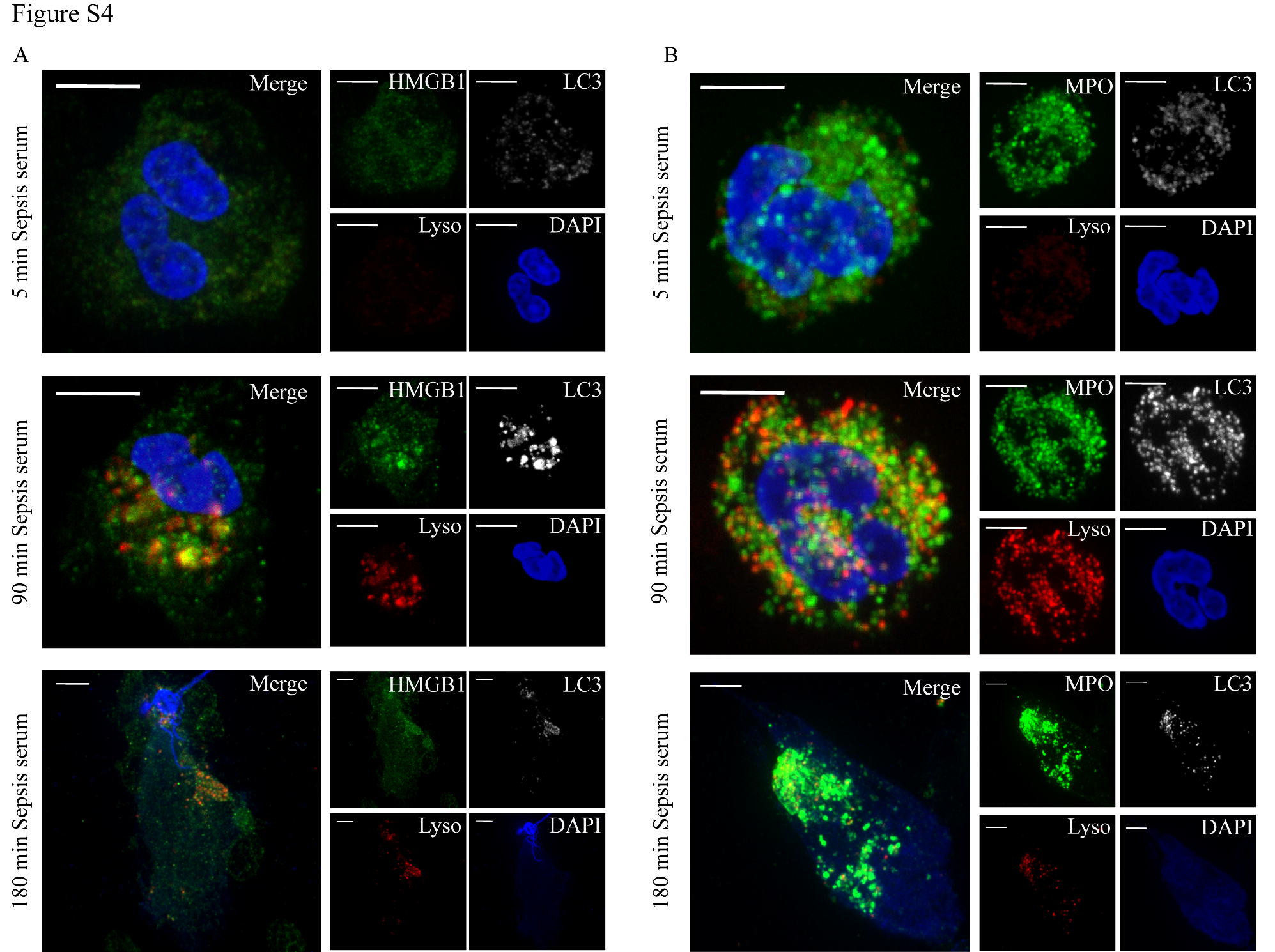

Supplement: Figure S4 — Localization of HMGB1 or MPO in late autophagosomes in control neutrophils treated septic serum. Control neutrophils were treated at different time points with septic serum and the colocalization of HMGB1 (A) and MPO (B) with autophagolysosomes was assessed. Autophagolysosomes were visualized by confocal microscopy as LC3B and LysoTracker double positive structures (z stack analysis, 0.3 µm per plane). (DNA labeled with DAPI; blue, anti-LC3B mAb; white, anti-MPO or anti-HMGB1 mAB; green, LysoTracker; red) (original magnification 1000×). One out of three independent experiments is shown. Scale bar represents 5 µM. (TIF) [file pone.0045427.s004.tif]

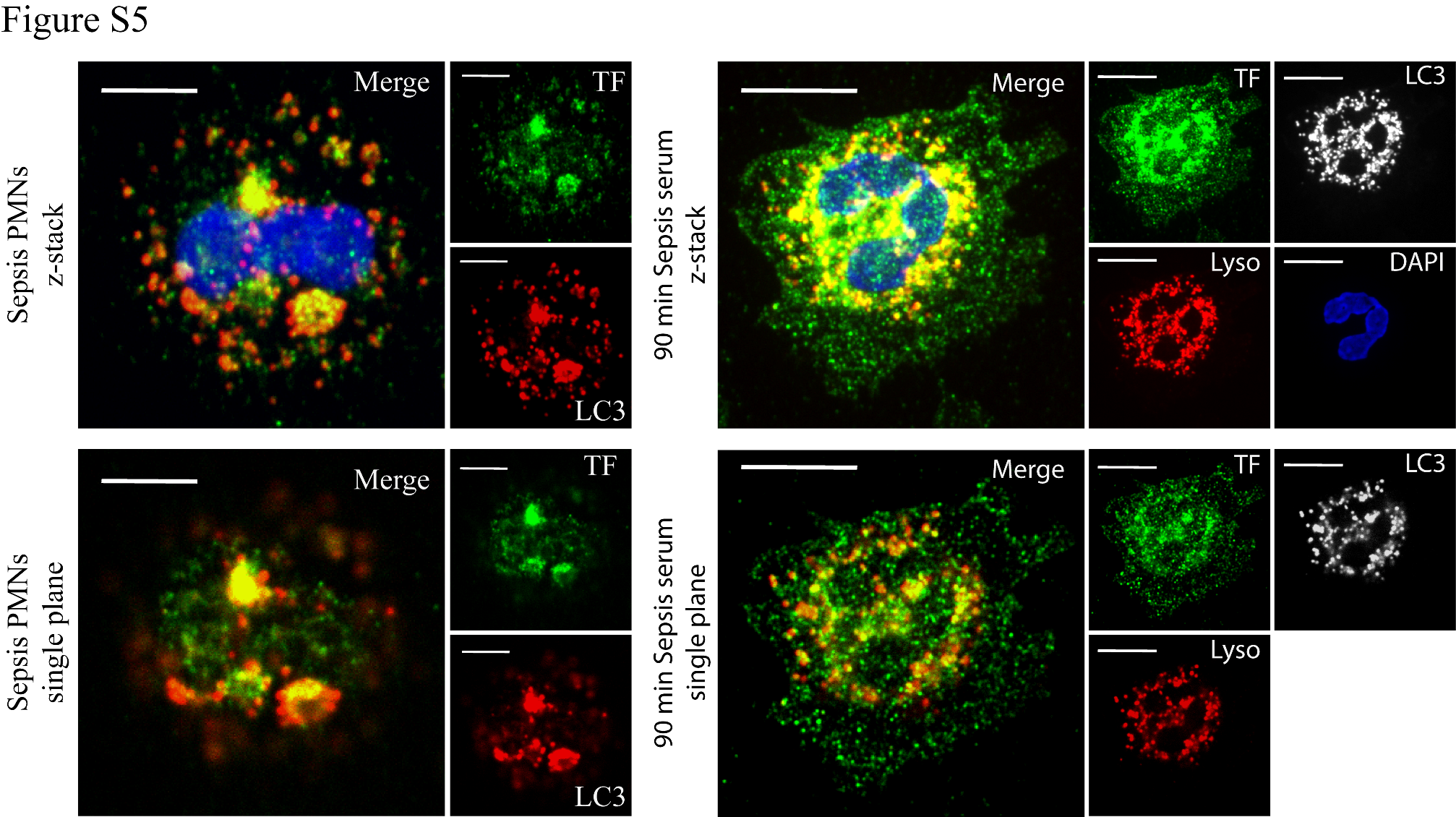

Supplement: Figure S5 — Comparison of z-stack with single plane analysis in colocalization studies. z-axies and Single Plane analysis of TF and LC3 in sepsis PMNs or control PMNs treated with sepsis serum. Scale bar represents 5 µM. (TIF) [file pone.0045427.s005.tif]
